# Decolorization of Bromophenol Blue by Free and Immobilized Crude Extracellular Laccase Preparation from *Bjerkandera adusta* TMF1 Produced on Agro-Industrial Residues

**DOI:** 10.3390/jof12070531

**Published:** 2026-07-19

**Authors:** Nevena Ilić, Jelena Lađarević, Katja Vasić, Maja Leitgeb, Željko Knez, Suzana Dimitrijević-Branković, Katarina Mihajlovski

**Affiliations:** 1 Innovation Centre of Faculty of Technology and Metallurgy, Karnegijeva 4, 11120 Belgrade, Serbia; nilic@tmf.bg.ac.rs; 2 Faculty of Technology and Metallurgy, University of Belgrade, Karnegijeva 4, 11000 Belgrade, Serbia; jmirkovic@tmf.bg.ac.rs (J.L.); suzana@tmf.bg.ac.rs (S.D.-B.); 3 Faculty of Chemistry and Chemical Engineering, University of Maribor, Smetanova 17, SI-2000 Maribor, Slovenia; katja.vasic@um.si (K.V.); maja.leitgeb@um.si (M.L.); zeljko.knez@um.si (Ž.K.); 4 Faculty of Medicine, University of Maribor, Taborska ulica 8, SI-2000 Maribor, Slovenia

**Keywords:** white rot fungus, laccase, enzyme production, agroindustrial waste, immobilization, biodegradation, decolorization, synthetic dyes

## Abstract

Synthetic dyes released from industrial effluents represent an important environmental challenge due to their persistence and toxicity. In this study, for the first time, a crude extracellular laccase preparation from *Bjerkandera adusta* TMF1 produced by solid-state fermentation on a wheat/barley bran mixture was immobilized onto glutaraldehyde-activated alginate beads and applied for mediator-free Bromophenol Blue (BPB) decolorization. The produced laccase showed high activity (51.51 IU/mL; 128.77 IU/g dry substrate), while immobilization efficiency reached 98.84% with 88.39% residual activity under optimal immobilization conditions. FTIR and SEM analyses indicated successful enzyme immobilization and structural changes in the alginate matrix. Under optimal conditions (50 mg/L BPB, pH 5, 30 °C), free and immobilized crude extracellular laccase preparation achieved 73.02% and 78.12% decolorization within 1 h, respectively, without the addition of synthetic redox mediators. The immobilized preparation retained decolorization ability during repeated use, maintaining more than 50% decolorization efficiency after the third cycle. HPLC analysis indicated changes in the chromatographic profile of BPB after enzymatic treatment, while phytotoxicity and antimicrobial assays suggested reduced toxicity of the treated samples toward the tested organisms. These results demonstrate that a crude extracellular laccase preparation produced by *B. adusta* TMF1 can serve as an efficient immobilized biocatalyst for Bromophenol Blue decolorization while supporting the sustainable valorization of agro-industrial residues for low-cost enzyme production.

## 1. Introduction

Synthetic dyes released from industrial effluents represent an environmental concern due to their persistence, toxicity, and resistance to conventional wastewater treatment [1,2,3]. Triphenylmethane dyes are particularly problematic due to their complex aromatic structures and potential toxic effects on living organisms [2,3]. Conventional treatment approaches, such as adsorption, coagulation, membrane filtration, and chemical oxidation, are often associated with high costs, incomplete pollutant removal, and the generation of secondary pollutants [3,4]. Consequently, enzymatic biodegradation has attracted increasing attention as an alternative approach for dye removal [1]. Among oxidative enzymes, fungal laccases (EC 1.10.3.2) are of particular interest due to their broad substrate specificity and ability to oxidize various aromatic compounds and recalcitrant pollutants [4,5].

White-rot fungi (WRF) are recognized as efficient producers of extracellular ligninolytic enzymes, including laccases with significant biotechnological potential [6,7]. In addition, solid-state fermentation (SSF) using lignocellulosic agro-industrial residues is considered an economical and efficient strategy for fungal enzyme production [8]. In contrast to submerged fermentation (SmF), which is often associated with high water consumption, lower enzyme concentrations, and increased downstream processing requirements, SSF offers several advantages, including the utilization of low-cost renewable substrates, reduced water and energy demands, lower operational costs, and a decreased risk of microbial contamination. Moreover, SSF provides conditions similar to the natural habitat of filamentous fungi, thereby promoting fungal growth and often enhancing enzyme production and stability [9]. Wheat bran (WB) and barley bran (BB) are abundant agricultural by-products rich in cellulose, hemicellulose, lignin, proteins, and minerals [10,11]. WB has frequently been used as a substrate for laccase production by different fungal species such as *Trametes versicolor* and *Pleurotus ferulae* [12,13], whereas barley bran remains less investigated. *Bjerkandera adusta* is known to produce several ligninolytic enzymes, including manganese peroxidase (MnP), lignin peroxidase (LiP), aryl alcohol oxidase (AAO), versatile peroxidase (VP) and dye-decolorizing peroxidases (DyPs), which are involved in lignin degradation and dye decolorization processes [14,15,16,17]. Previous studies have reported the production of MnP and LiP by *B. adusta* cultivated on lignocellulosic substrates such as wood meal supplemented with wheat bran [14], while AAO and MnP were identified as major extracellular oxidative enzymes under specific cultivation conditions [15]. These enzymes contribute to the degradation of various synthetic dyes, and *B. adusta* has demonstrated high decolorization efficiencies for textile dye effluents [16]. In addition, co-cultivation strategies have also been reported to enhance oxidative enzyme production in *B. adusta*, resulting in increased peroxidase activity and improved dye decolorization efficiencies (up to ~90%) compared to monocultures, highlighting the importance of interspecific fungal interactions for enhanced bioremediation performance [17]. However, information on laccase production by fungi of the genus *Bjerkandera* remains limited, and several studies reported low or inconsistent laccase activity in some *B. adusta* strains [18,19]. Although *Bjerkandera* species have been associated with dye degradation processes, reports describing mediator-free Bromophenol Blue (BPB) decolorization using *B. adusta* laccase are still lacking. BPB is a commonly used triphenylmethane dye characterized by high persistence in aquatic environments, toxicity, phototoxicity toward aquatic organisms, and a strong potential for bioaccumulation [20].

Despite the significant catalytic potential of laccases, the application of soluble enzymes in industrial processes is often limited by low operational stability, poor reusability, and sensitivity to environmental conditions [21,22]. Enzyme immobilization is widely recognized as an effective strategy for overcoming these limitations because it can improve enzyme stability and enable repeated use of the biocatalyst [22,23]. Among different immobilization supports, alginate beads are widely used due to their biocompatibility, biodegradability, low toxicity, and simple preparation procedure [24,25]. In addition, glutaraldehyde activation can improve enzyme attachment and immobilization efficiency by promoting covalent interactions between the enzyme and support material [25].

**Figure 1:**
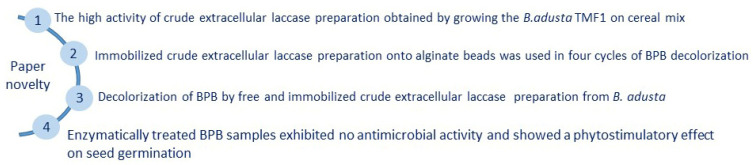
Schematic representation of summarized novel aspects of the study.

In this study, crude extracellular laccase preparation produced by *B. adusta* TMF1 during SSF on a WB/BB mixture was immobilized onto glutaraldehyde-activated alginate beads and applied for BPB decolorization. To the best of our knowledge, this is the first report on the application of immobilized crude extracellular laccase preparation from *B. adusta* TMF1 produced on a WB/BB mixture for mediator-free BPB decolorization. The resulting immobilized biocatalyst was characterized by FTIR, SEM, and kinetic analyses, while its decolorization efficiency and reusability were evaluated under different operational conditions. In addition, changes in the chromatographic profile of BPB after enzymatic treatment were investigated by HPLC, and the eco-safety of treated BPB samples was assessed using phytotoxicity and antimicrobial assays. All novel aspects of the study are summarized in Figure 1.

## 2. Materials and Methods

### 2.1. Chemicals

ABTS (2,2′-azino-bis(3-ethylbenzothiazoline-6-sulfonic acid)), sodium alginate, glutaraldehyde (GA), calcium chloride (CaCl_2_), and bovine serum albumin (BSA) were obtained from Sigma-Aldrich (Merck KGaA, Darmstadt, Germany). Bromophenol Blue (BPB, St. Louis, MO, USA) was used as a model dye for decolorization experiments. Malt extract broth, de Man, Rogosa and Sharpe broth (MRS broth), tryptic soy broth (TSB), agar, and yeast extract were purchased from Torlak (Belgrade, Serbia). WB and BB were purchased from a retail store, Belgrade, Serbia.

### 2.2. Microorganisms and Plant Material

The white-rot fungus *B. adusta* TMF1 (MW327505) was used for laccase production [26]. *Lactobacillus rhamnosus* ATCC 7469, *Candida albicans* ATCC 10259, and waste brewer’s yeast *Saccharomyces cerevisiae* [27], used in antimicrobial assays, were obtained from the culture collection of the Department of Biochemical Engineering and Biotechnology, Faculty of Technology and Metallurgy, University of Belgrade, Serbia. Wheat seeds (*Triticum aestivum*) used for phytotoxicity assays were commercially obtained in Belgrade, Serbia.

### 2.3. Inoculum Preparation

*B. adusta* TMF1 was maintained on malt agar plates (malt extract broth 20 g/L and agar 15 g/L) at 30 °C. Fungal inoculum preparation was performed according to the procedure described in our previous study [21]. *L. rhamnosus* ATCC 7469 was cultivated in MRS broth at 37 °C, while *C. albicans* ATCC 10259 was cultivated in TSB supplemented with 0.6% yeast extract at 37 °C. Waste brewer’s yeast *S. cerevisiae* was grown in malt extract broth at 30 °C.

### 2.4. Laccase Production Under Solid-State Fermentation

Laccase production was carried out by SSF using a WB/BB mixture (1:1, *w*/*w*) as substrate. The cereal mixture was adjusted to 70% moisture content, autoclaved at 121 °C for 20 min in 300 mL Erlenmeyer flasks, and inoculated with eight mycelial discs (1 × 1 cm) of *B. adusta* TMF1. Fermentation was performed at 30 °C under dark conditions for 9 days. Crude laccase extraction, including ammonium sulfate precipitation (70% saturation) and membrane concentration, was carried out according to the procedure described in our previous study [28]. The resulting enzyme preparation is hereafter referred to as the crude extracellular laccase preparation throughout this manuscript.

### 2.5. Laccase Immobilization onto Alginate Beads

Alginate beads were prepared using 5% (*w*/*v*) sodium alginate dissolved in acetate buffer (0.1 M, 10 mL, pH 5). The alginate solution was stirred for 1 h and added dropwise into 0.1 M CaCl_2_ solution to form beads, which were further stabilized in the same solution for 2 h.

Activation of alginate beads was performed according to Hu et al. [29] with minor modifications. Briefly, the beads were incubated with different GA concentrations (2–10%, *v*/*v*) at a bead:GA solution ratio of 1:10 for 30, 60, 90, and 120 min in order to determine the optimal activation conditions. The activation procedure was carried out at 25 °C under orbital shaking (150 rpm). After activation, the beads were washed three times with acetate buffer (0.1 M, 15 mL, pH 5).

**Figure 2:**
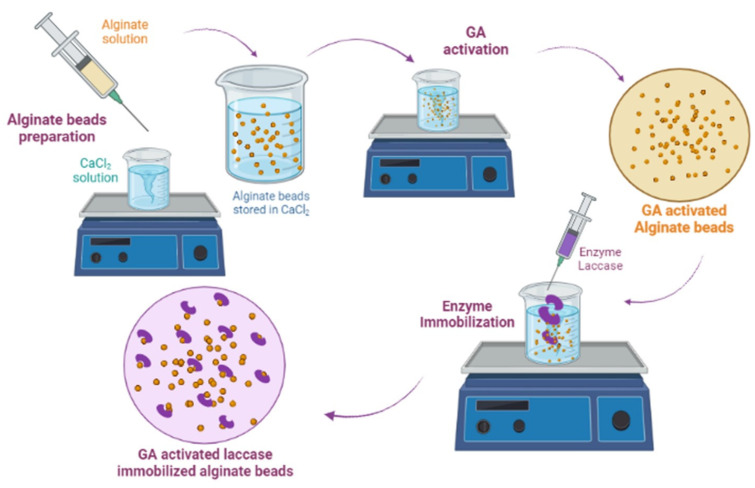
Schematic representation of the immobilization of the crude extracellular laccase preparation onto GA-activated alginate beads.

For laccase immobilization, GA-activated alginate beads were incubated with the crude extracellular laccase preparation from *B. adusta* TMF1 for different time intervals (30, 60, 90, and 120 min) at 25 °C under orbital shaking (150 rpm). In each experiment, 5 mL of crude extracellular laccase preparation (pH 5) was mixed with three GA-activated alginate beads. Following immobilization, the beads were washed three times with acetate buffer (0.1 M, 15 mL, pH 5) and used for laccase activity determination. The immobilization procedure is schematically presented in Figure 2.

### 2.6. Laccase Activity Assay

Laccase activity was determined spectrophotometrically by monitoring the oxidation of ABTS at 420 nm according to Primožič et al. [30]. The reaction mixture contained 0.2 mL of 1 mM ABTS, 0.6 mL of 0.1 M sodium acetate buffer (pH 5), and 0.4 mL of crude extracellular laccase preparation. Enzyme activity is expressed as IU/mL.

Laccase activity (IU/mL) was calculated according to Equation (1):

(1) IU/mL = ((A_sample_/t) × 1.2 × d_f_)/(3.6 × V_enzyme_)

where A_sample_ is the absorbance of the sample, t is the reaction time (1 min), 1.2 is the total volume of the reaction mixture (mL), df is the dilution factor, 3.6 is the extinction coefficient of ABTS, and V_enzyme_ is the volume of the crude extracellular laccase preparation in the reaction mixture (mL) [30].

For immobilized crude extracellular laccase preparation, the assay was performed under the same conditions using three alginate beads. All experiments were performed in triplicates with standard deviation less than ± 3% and all results represent the mean value of three measurements.

To further characterize the crude extracellular laccase preparation used for immobilization, MnP and LiP activities were additionally determined according to the methods described by Jović et al. [31]. In addition, SDS–PAGE analysis was performed to assess the protein profile of the crude extracellular enzyme preparation. SDS–PAGE was performed on a 10% polyacrylamide resolving gel with a 4% polyacrylamide stacking gel using a mini vertical electrophoresis system (SE260 Mighty Small II Deluxe Mini Vertical Protein Electrophoresis Unit; Hoefer Inc., Holliston, MA, USA). Protein samples were prepared by mixing 100 μL of the crude extracellular laccase preparation with 50 μL of sample loading buffer (0.5 M Tris–HCl buffer, 99% glycerol, 10% SDS, bromophenol blue, and β-mercaptoethanol added immediately before use) followed by heating in a boiling water bath (95 º) for 5 min before loading onto the gel. Prepared samples were loaded onto gel along with pre-strained protein marker (10–250 kDa, Spectra Multicolor Broad Range Protein Ladder; Thermo Scientific, Waltham, MA, USA. The separation was performed with running buffer (3 g Tris, 14.4 g glycine, and 10 mL of 10% SDS, diluted to a final volume of 1 L with distilled water). Electrophoresis was carried out at 80 V until the samples entered the resolving gel, after which the voltage was increased to 150 V and maintained until completion (approximately 90 min). Proteins were visualized by staining with a solution of Coomassie Brilliant Blue R250 (CBB) (0.1% CBB, 10% acetic acid 50% methanol) followed by destaining with a solution of 5% methanol and 7% acetic acid overnight.

### 2.7. Immobilization Efficiency and Residual Activity Determination

Protein concentration was determined by the Bradford method using BSA as a standard, with absorbance measured at 595 nm. In the case of immobilized crude extracellular laccase preparation, the protein concentration was determined in the supernatants collected after rinsing the alginate beads. All experiments were performed in triplicate, and the results are presented as mean values ± standard deviation.

Immobilization efficiency (IE) was calculated according to Equation (2):

(2) Immobilization efficiency (%) = ((c_i_ − c_s_)/c_i_) × 100

where c_i_ is the protein concentration of the crude extracellular laccase preparation [mg/mL], and c_s_ is the protein concentration of the supernatant after rinsing [mg/mL] as described in 2.5.

Residual activity was calculated according to Equation (3) [30]:

(3) A (%) = (Laccase activity of the immobilized preparation/Laccase activity of free preparation) × 100

where the activities refer to the measured laccase activity of the free and immobilized crude extracellular laccase preparations.

### 2.8. Determination of the Kinetics Parameters K_m_ and V_max_

The Michaelis–Menten constant (K_m_) and maximum reaction velocity (V_max_) of free and immobilized crude extracellular laccase preparation were determined according to Primožič et al. [32] using different ABTS concentrations (1–5 mM). Kinetic parameters were calculated according to the Michaelis–Menten model using Lineweaver–Burk plots [33]. All experiments were performed in triplicate, and the results are presented as mean values ± standard deviation.

### 2.9. Reusability of Immobilized Crude Extracellular Laccase Preparation

The reusability of immobilized crude extracellular laccase preparation was evaluated during 11 consecutive reaction cycles using ABTS as substrate. After each cycle, the alginate beads were washed three times with 1 mL of sodium acetate buffer (0.1 M, pH 5) and reused in the subsequent reaction cycle. The residual activity after each cycle was calculated relative to the initial activity of the immobilized crude extracellular laccase preparation, which was considered as 100%.

### 2.10. SEM Analysis

The surface morphology of alginate beads before and after immobilization of the crude extracellular laccase preparation was examined using scanning electron microscopy (SEM) (Field Emission Scanning Electron Microscopy, TESCAN, Kohoutovice, Czech Republic) (Tescan FE-SEM Mira 3 XMU, operated at 20 kV) (Polaron SC503, Fisons Instruments, UK). Prior to SEM analysis, all samples were coated with a gold alloy using a sputter coater (Polaron SC503, Fisons Instruments).

### 2.11. FT-IR Analysis

FT-IR spectra of the crude extracellular laccase preparation, alginate beads, and immobilized crude extracellular laccase preparation onto alginate beads were recorded using a Nicolet™ iS™ 10 FT-IR spectrometer (Thermo Fisher Scientific) equipped with a Smart iTR™ attenuated total reflectance (ATR) accessory. Spectra were recorded in the range of 400–4000 cm^−1^ using 32 scans per sample.

### 2.12. Decolorization of BPB

The effect of dye concentration on BPB decolorization was evaluated using four different dye concentrations (25–100 mg/L) treated with crude extracellular laccase preparation from *B. adusta* TMF1 (51.51 IU/mL). Based on the obtained results, a BPB concentration of 50 mg/L was selected for further experiments.

To determine the optimal pH and temperature for BPB decolorization, free and immobilized crude extracellular laccase preparations were separately tested at different temperatures (30–50 °C) and pH values (3–7). For the free enzyme preparation, 250 µL of crude extracellular laccase preparation (51.51 IU/mL) was added to 10 mL of BPB solution. For the immobilized enzyme preparation, 30 alginate beads containing immobilized crude extracellular laccase preparation (50.78 IU/mL) were added to the reaction mixture. Decolorization experiments were performed for 60 min, and aliquots were collected every 15 min during the reaction.

After each decolorization cycle, the supernatants were collected and analyzed for protein concentration and residual laccase activity in order to assess potential enzyme leakage from the immobilization support.

BPB decolorization was determined spectrophotometrically by measuring absorbance at 592 nm. The percentage of decolorization was calculated according to Equation (4):

(4) BPB decolorization (%) = ((Absorbance_initial_ − Absorbance_time_)/Absorbance_initial_) × 100

### 2.13. HPLC Analytical Procedure

Samples were analyzed using a Dionex Ultimate 3000 ThermoScientific (Voltam, Waltham, MA, USA) HPLC system with a PerkinElmer C18 reversed-phase column, 150 mm × 4.6 mm, 5 μm. The following mobile phases (A) H_2_O:HCOOH = 100:0.1 (*v*/*v*)% and (B) acetonitrile were used for the analysis of reaction mixture samples. An isocratic elution with 80% acetonitrile was used. The flow rate of the mobile phase in all analyses was 1 mL/min, and the column was thermostated at 30 °C. The injection volume of the samples was 40 µL. Changes in the chromatographic profile of BPB after enzymatic treatment were monitored at 592 nm and 254 nm.

### 2.14. Phytotoxicity Assay

Phytotoxicity assays were performed to evaluate the effects of BPB and its enzymatically treated products using wheat seeds (*T. aestivum*) as a model plant system, according to the procedure described by Ilić et al. [28].

### 2.15. Antimicrobial Activity Assay

The effects of BPB and its enzymatically treated products on *C. albicans*, *S. cerevisiae*, and *L. rhamnosus* were evaluated according to the procedure described by Ilić et al. [28].

## 3. Results

### 3.1. Laccase Production

In the present study, for the first time, a mixture of WB and BB was shown to be an effective substrate for laccase production by *B. adusta* TMF1. Under applied SSF conditions (WB:BB ratio 1:1, moisture content 70%, 30 °C), the strain produced a maximum laccase activity of 51.51 IU/mL (128.77 IU/g) after 9 days of cultivation. To further characterize the obtained crude extracellular laccase preparation, MnP and LiP activities were additionally determined. Neither MnP nor LiP activity was detected under the applied cultivation and enzyme preparation conditions. The protein profile of the crude extracellular laccase preparation was additionally analyzed by SDS-PAGE. As shown in Supplementary Figure S1, multiple protein bands were observed, confirming the heterogeneous composition of the crude extracellular laccase preparation. A prominent protein band was detected in the molecular weight region around 50 kDa, which is consistent with the reported molecular weight of many fungal laccases [7].

### 3.2. Immobilization of the Crude Extracellular Laccase Preparation onto Alginate Beads

The crude extracellular laccase preparation was immobilized onto Ca-alginate beads previously activated with GA. The effects of GA activation time, GA concentration, and immobilization time on immobilization efficiency (IE) and residual laccase activity were evaluated.

**Figure 3:**
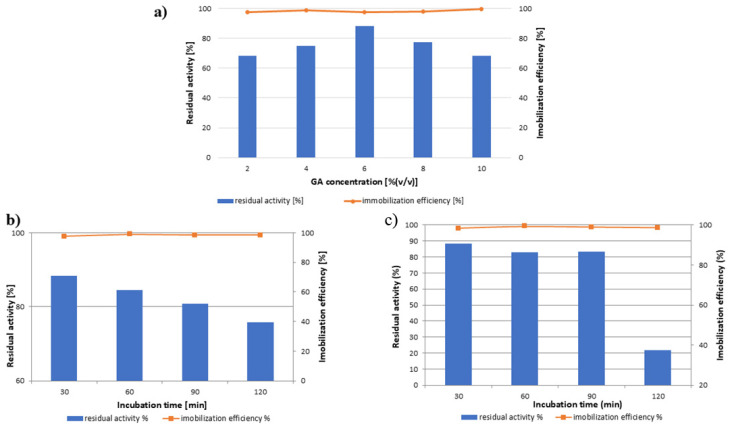
Optimization of incubation time of alginate beads with GA (**a**), optimal GA concentration for immobilization of the crude extracellular laccase preparation (**b**) and incubation time of GA-activated beads with laccase (**c**).

Different activation times (30, 60, 90, and 120 min) were examined using 6% (*v*/*v*) GA. The highest IE (98.84%) and residual activity (88.39%) were obtained after 30 min of activation (Figure 3a). Increasing the activation time resulted in a gradual decrease in residual laccase activity, whereas IE remained above 98% under all tested conditions.

The effect of GA concentration was investigated in the range of 2–10% (*v*/*v*) using an activation time of 30 min (Figure 3b). The highest residual activity and IE were obtained with 6% (*v*/*v*) GA. Both lower and higher GA concentrations led to reduced residual activity, while IE remained consistently high.

The effect of immobilization time was evaluated using incubation periods of 30, 60, 90, and 120 min (Figure 3c). The highest residual activity (88.39%) was achieved after 30 min of immobilization. Further prolongation of the immobilization time resulted in decreased enzyme activity, whereas IE remained above 98% under all tested conditions.

### 3.3. Kinetics Parameters K_m_ and V_max_

Kinetic parameters (Km and Vmax) of free and immobilized crude extracellular laccase preparation were determined using different ABTS concentrations (1–5 mM). Based on the measured laccase activity, the free preparation exhibited a Km value of 86 µM, which increased to 376 µM after immobilization. In contrast, the corresponding Vmax decreased from 549 µM/min to 153 µM/min following immobilization (Table 1).

### 3.4. Reusability of the Immobilized Crude Extracellular Laccase Preparation

The operational stability of the alginate-immobilized crude extracellular laccase preparation from *B. adusta* TMF1 was evaluated through repeated cycles of ABTS oxidation, as reusability is an important parameter for potential industrial applications.

**Figure 4:**
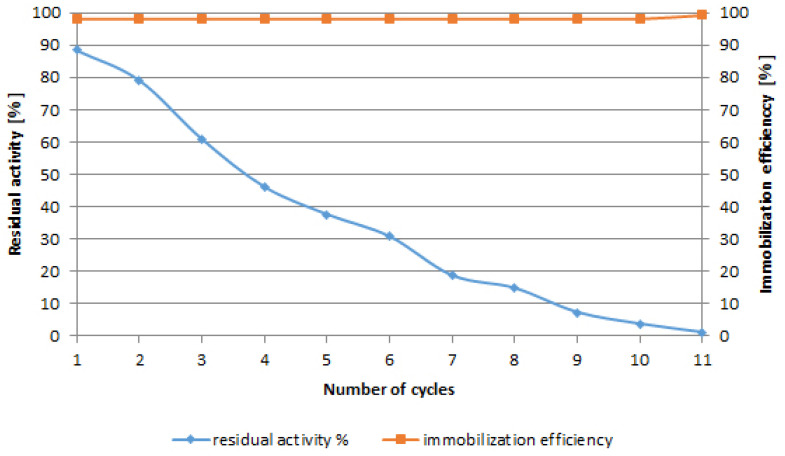
Reusability of the immobilized crude extracellular laccase preparation based on laccase activity measured using ABTS.

The immobilized biocatalyst retained detectable laccase activity during 11 consecutive cycles (Figure 4).

The highest residual activity was observed during the first cycle (88.39%), followed by 79.13% and 61.11% after the second and third cycles, respectively. The immobilized biocatalyst retained approximately 46.1% activity in the fourth cycle, while residual laccase activity remained above 30% during the fifth and sixth cycles (37.56% and 31.00%, respectively). In the subsequent cycles, residual laccase activity progressively decreased to below 20%.

### 3.5. Surface Morphology of the Immobilized Crude Extracellular Laccase Preparation

The surface morphology of Ca-alginate beads before and after immobilization of the crude extracellular laccase preparation was analyzed by SEM. SEM micrographs revealed morphological alterations after enzyme immobilization [34]. Prior to immobilization, the beads exhibited a predominantly spherical shape with an approximate diameter of 1.5 mm. After immobilization, slight deformation of the bead structure toward an ellipsoidal shape was observed.

**Figure 5:**
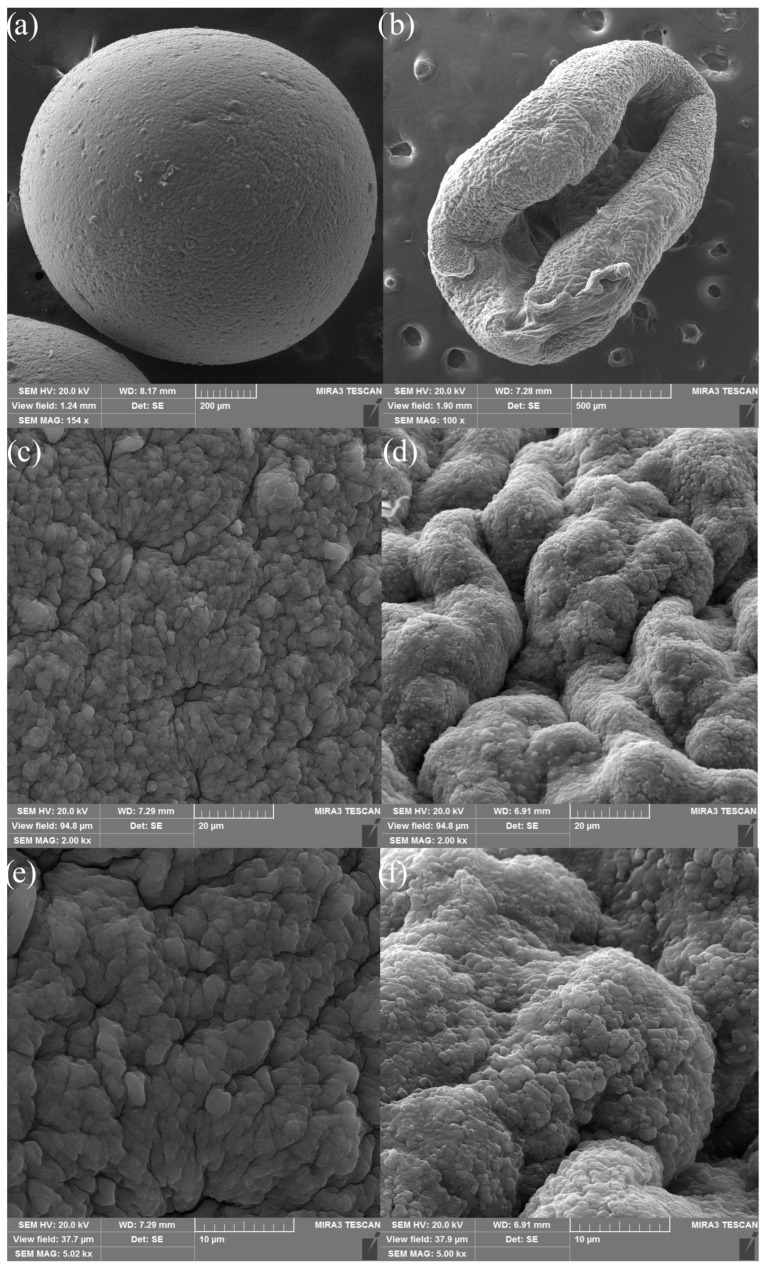
SEM images of alginate beads without immobilized crude extracellular laccase preparation (control sample) and with immobilized crude extracellular laccase preparation: (**a**,**b**) 100 ×; (**c**,**d**) 2000 ×; and (**e**,**f**) 5000 ×.

Control alginate beads without immobilized crude extracellular laccase preparation (Figure 5a,c,e) showed a relatively compact surface structure, whereas beads containing the immobilized crude extracellular laccase preparation (Figure 5b,d,f) exhibited larger and more irregular surface pores. In addition, crystalline structures were observed on the surface of immobilized beads, possibly associated with residual ammonium sulfate used during laccase precipitation.

### 3.6. FT-IR Analysis

**Figure 6:**
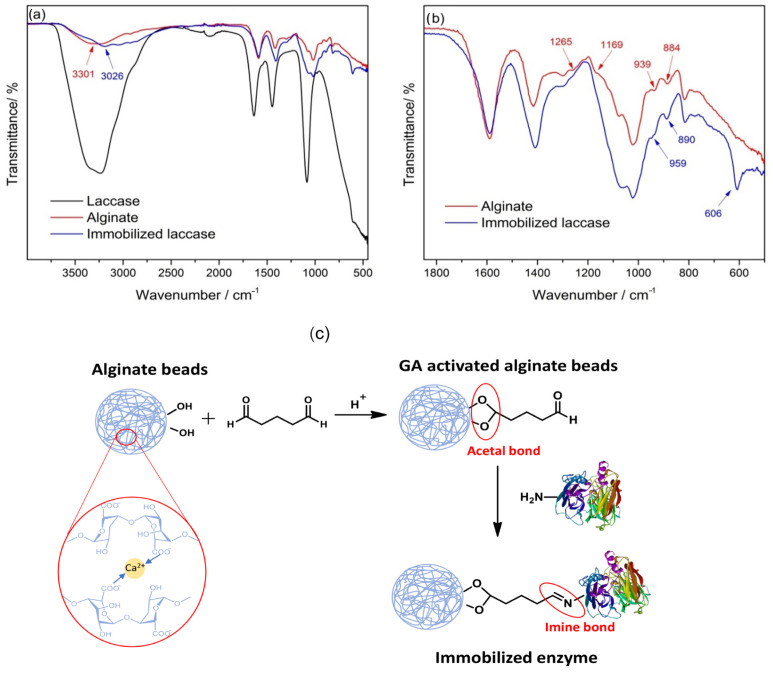
FTIR spectra of control alginate beads (**a**) and alginate beads with immobilized crude extracellular laccase preparation (**b**), and schematic representation of immobilization of crude extracellular laccase preparation on alginate beads (**c**).

FTIR spectroscopy was used to investigate structural changes after immobilization of the crude extracellular laccase preparation onto GA-activated alginate beads. FTIR spectra of the free crude extracellular laccase preparation, alginate beads, and alginate beads containing the immobilized crude extracellular laccase preparation are presented in Figure 6.

In the spectrum of the free crude extracellular laccase preparation, a broad band in the range of 3200–3400 cm^−1^ was observed. For alginate beads, a characteristic peak at 3301 cm^−1^ was detected. After immobilization, this peak was no longer observed, while a new peak appeared at 3026 cm^−1^.

Additional spectral modifications were also recorded after immobilization. The peaks at 1265 cm^−1^ and 1169 cm^−1^ were absent in the immobilized sample. Moreover, shifts of peaks from 939 cm^−1^ and 884 cm^−1^ to 959 cm^−1^ and 890 cm^−1^, respectively, were observed. A new band at 606 cm^−1^ appeared in the immobilized preparation.

### 3.7. BPB Decolorization

The effects of dye concentration, pH, and temperature on BPB decolorization by the crude extracellular laccase preparation from *B. adusta* TMF1 were investigated.

**Figure 7:**
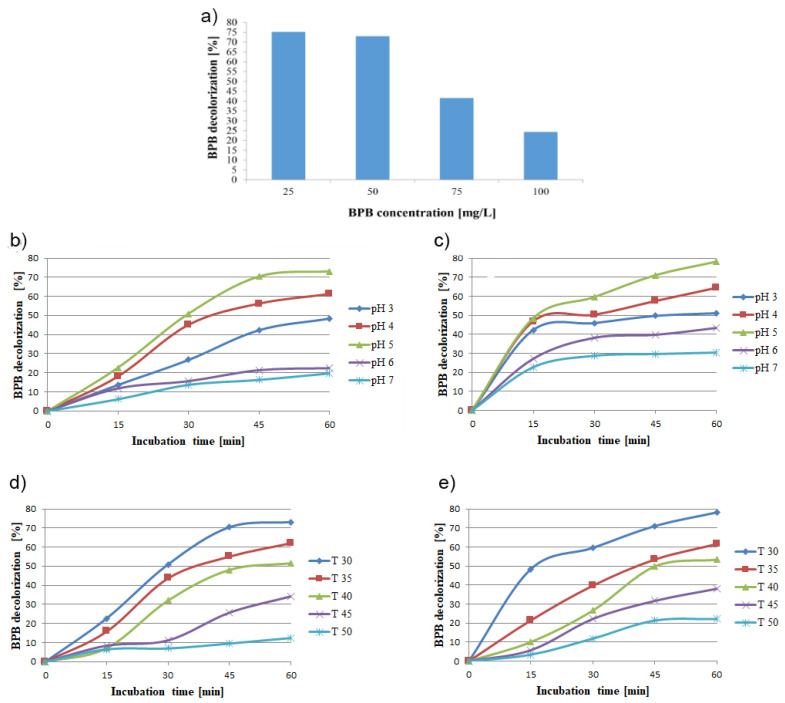
Optimization of BPB decolorization: dye concentration (**a**); effect of different pHs on decolorization with free crude extracellular laccase preparation (**b**); effect of different pHs on decolorization with immobilized crude extracellular laccase preparation (**c**); effect of different temperatures on decolorization with free crude extracellular laccase preparation (**d**); and effect of different temperatures on decolorization with immobilized crude extracellular laccase preparation (**e**).

Different BPB concentrations (25–100 mg/L) were treated with free crude extracellular laccase preparation (51.51 IU/mL) at pH 5 and 30 °C for 60 min. As shown in Figure 7a, the highest decolorization efficiency (75.21%) was obtained at 25 mg/L BPB, while slightly lower decolorization (73.02%) was achieved at 50 mg/L under the same conditions. A further increase in dye concentration resulted in decreased decolorization efficiency, reaching 24.32% at 100 mg/L BPB.

The effects of pH (3–7) and temperature (30–50 °C) on BPB decolorization were further evaluated using free and alginate-immobilized crude extracellular laccase preparation at a constant BPB concentration of 50 mg/L. Both the free and immobilized preparation exhibited the highest decolorization efficiency at pH 5 and 30 °C (Figure 7b–e).

**Figure 8:**
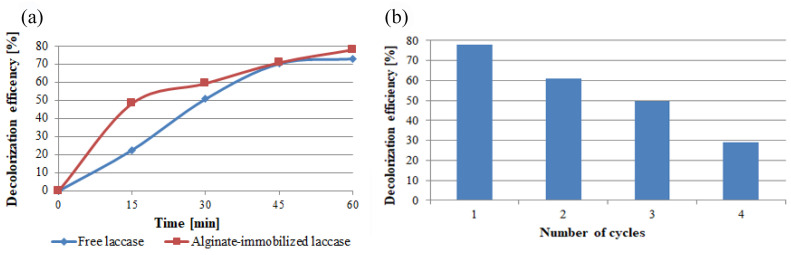
Time-course of BPB decolorization by free and immobilized crude extracellular laccase preparation (**a**) and reusability of the immobilized crude extracellular laccase preparation during BPB decolorization (**b**).

Under optimal conditions, the free crude extracellular laccase preparation achieved 73.02% BPB decolorization after 60 min, whereas the immobilized preparation reached 78.12% under the same conditions (Figure 8a). The immobilized preparation also showed faster decolorization during the initial reaction period, achieving 48.42% and 59.51% after 15 and 30 min, compared with 22.65% and 50.88% for free laccase.

The immobilized crude extracellular laccase preparation retained its activity over four consecutive BPB decolorization cycles, with efficiencies of 61.1%, 50.21%, and 29.21% in the second, third, and fourth cycles, respectively (Figure 8b).

### 3.8. HPLC Analysis

Changes in the chromatographic profile of BPB after enzymatic treatment with the crude extracellular laccase preparation from *B. adusta* TMF1 were analyzed by HPLC using isocratic elution. UV detection was performed at 592 nm and 254 nm. As shown in Supplementary Figure S2a,b, the chromatographic profiles of untreated and treated samples showed noticeable differences at 254 nm, whereas similar peaks were observed at 592 nm. In the treated sample, two additional smaller peaks appeared at 254 nm, suggesting the formation of transformation products during enzymatic treatment.

### 3.9. Phytotoxicity

Wheat seeds (*T. aestivum*) were used as a model plant system to evaluate the phytotoxicity of BPB and enzymatically treated samples. The obtained results showed that neither BPB nor the treated samples inhibited seed germination. The number of germinated seeds was 14 and 15 for BPB and treated samples, respectively (Supplementary Figure S3).

The relative germination value (RGV) was 93.33% for BPB and 100% for the treated samples, while the relative root length value (RLV) was 76.7% and 130.5%, respectively. Based on these parameters, the germination index (GI) was calculated as 71.6% for BPB and 130.5% for the treated samples.

### 3.10. Antimicrobial Activity

The effects of BPB and BPB samples treated with the crude extracellular laccase preparation on *C. albicans*, *S. cerevisiae*, and *L. rhamnosus* were investigated (Table 2). BPB slightly reduced the number of viable *C. albicans* cells by 5.17% compared with the control sample, whereas no inhibitory effect toward *L. rhamnosus* was observed.

The enzymatically treated BPB samples showed no inhibitory effects toward the tested microorganisms. Slight increases in viable cell numbers were observed for all tested strains. In the case of *C. albicans* and *L. rhamnosus*, the increases were 1.8% and 2.12%, respectively. The number of viable *S. cerevisiae* cells in the presence of untreated BPB was 7.32% higher than in the control, while after enzymatic treatment this increase reached 8.77% (Table 2).

## 4. Discussion

Despite the recognized ligninolytic potential of white-rot fungi, information on laccase production by fungi belonging to the genus *Bjerkandera* remains lacking. Moreover, several studies have reported low or inconsistent laccase activity among different *B. adusta* strains, while some strains were even found to be incapable of producing detectable levels of laccase [18,19]. The maximum laccase activity produced by *B. adusta* TMF1 (51.51 IU/mL; 128.77 IU/g) compares favorably with that reported for several other *B. adusta* strains, although higher activities have also been reported under different cultivation conditions. For example, Tripathi et al. [15] reported a maximum laccase activity of 5.5 IU/L after 10 days of cultivation in a nutrient-rich medium. Similarly, Kang et al. [35] reported a laccase activity of 0.21 IU/mL for *B. adusta* SM46 cultivated on rice straw, whereas the highest activity reported for *B. adusta* TBB-03 grown on a lignocellulosic substrate was 211.4 IU/L (0.2114 IU/mL) [15]. In addition, some *B. adusta* strains were reported to exhibit low or negligible laccase activity [36,37].

Although direct comparison among studies is difficult due to differences in cultivation conditions, substrates, and activity assays, the relatively high laccase activity obtained for strain *B. adusta* TMF1 indicates its promising biocatalytic potential. Furthermore, the present results demonstrate that low-cost agro-industrial residues such as wheat bran and barley bran can serve as suitable substrates for laccase production by *B. adusta*, supporting their valorization for sustainable enzyme production processes.

It should be emphasized that *B. adusta* has been reported to produce a complex ligninolytic enzyme system, including laccase, MnP, LiP, VP, and other oxidative enzymes, depending on the fungal strain and cultivation conditions [14,15,16]. In the present study, however, only laccase activity was detected in the crude extracellular enzyme preparation obtained under the applied cultivation and extraction conditions, whereas MnP and LiP activities were not detected. Although the presence of other extracellular oxidative enzymes cannot be completely excluded, these findings suggest that laccase was the predominant detectable ligninolytic activity in the enzyme preparation used for the subsequent immobilization and dye decolorization experiments.

Given the high laccase activity achieved by *B. adusta* TMF1, the next step was to improve the applicability of the crude extracellular laccase preparation through immobilization. Enzyme immobilization is widely used to enhance enzyme stability, facilitate enzyme recovery and reuse, and improve process feasibility in industrial and environmental applications. In the present study, GA-activated Ca-alginate beads proved to be an efficient support for immobilization of the crude extracellular laccase preparation, resulting in immobilization efficiencies above 98% under all tested conditions. The highest immobilization efficiency (98.84%) and residual laccase activity (88.39%) were obtained after 30 min of support activation with 6% (*v*/*v*) GA and 30 min of immobilization. Although enzyme binding remained highly efficient under all tested conditions, prolonged activation and immobilization times led to a gradual decrease in residual activity [38]. This behavior may be attributed to excessive crosslinking, which may restrict enzyme flexibility and alter the conformation of the active site, thereby reducing catalytic performance [38].

The results obtained for different GA concentrations further support this explanation. While 6% (*v*/*v*) GA provided the best balance between enzyme binding and catalytic activity, both lower and higher GA concentrations resulted in reduced residual activity despite maintaining high immobilization efficiency. Similar observations were reported by Ortega et al. [39], who demonstrated that GA activation improved immobilization performance compared with non-activated alginate beads. Likewise, other studies have shown that excessive GA concentrations can adversely affect enzyme activity due to structural modifications caused by over-crosslinking [25,29,39]. Comparable effects have also been observed during the immobilization of laccase from *Trametes pubescens* MB89 and xylanase on GA-activated alginate supports [25,29].

The incubation time of crude extracellular laccase preparation with GA-activated beads significantly influenced the activity of the immobilized enzyme. Prolonged contact between the enzyme and the activated support may lead to excessive cross-linking, which can alter enzyme conformation, reduce the flexibility of the active site, and consequently decrease catalytic activity [40]. However, the contact time between laccase from *B. adusta* TMF1 and GA-activated alginate beads did not affect the IE. Ortega et al. [39] reported similar results for neutrase immobilization onto GA-activated alginate beads where the contact time between the enzyme and the support had no significant effect on the immobilization yield.

The consistently high immobilization efficiencies obtained in this study indicate strong binding of the crude laccase molecules to the activated alginate matrix, whereas the retention of nearly 90% of the initial activity under optimal conditions indicates that the immobilization procedure caused only minimal loss of catalytic function. These findings suggest that GA-activated Ca-alginate beads represent a suitable carrier for immobilization of crude extracellular *B. adusta* TMF1 laccase preparation and support their potential application in biocatalytic and wastewater treatment processes.

Following the successful immobilization of the crude extracellular laccase preparation by *B. adusta* TMF1 onto GA-activated Ca-alginate beads, the catalytic properties of the enzyme were further assessed by determining the kinetic parameters (Km and Vmax). Immobilization resulted in a pronounced change in kinetic behaviour, with some increase in Km accompanied by a marked reduction in Vmax compared to the free enzyme preparation. The higher Km value of the immobilized preparation reflects a lower apparent affinity for the substrate, which is commonly associated with mass transfer limitations and reduced accessibility of ABTS molecules to the active sites within the alginate matrix [39,41]. Similarly, the decrease in Vmax suggests a diminished maximal catalytic capacity, which may arise from conformational restrictions of the proteins responsible for the measured laccase activity, as well as from their interaction with the support following immobilization [41].

Comparative studies have shown that the effect of immobilization on laccase kinetics varies depending on the fungal source and immobilization system. For example, laccase from *T. versicolor* IBL-04 exhibited Km and Vmax values of 69.90 µM and 720.7 µM/min, respectively, while its alginate-immobilized form showed Km and Vmax values of 77 µM and 876.4 µM/min [42]. In contrast, Varga et al. [18] reported considerably lower kinetic values for alginate-immobilized *T. versicolor* laccase (Km = 26.43 µM; Vmax = 0.23 µM/min), further demonstrating that the effect of immobilization on enzyme kinetics strongly depends on the enzyme origin and the nature of the immobilization support.

These kinetic changes are closely related to the operational behavior of the immobilized system during repeated use. The gradual decline in residual activity observed during consecutive reaction cycles may be attributed to a combination of factors, including limited substrate diffusion into the alginate matrix, accumulation of reaction products on the bead surface, and partial enzyme inactivation over successive cycles. Despite this decrease in activity, immobilization efficiency remained consistently high (>98%) throughout all cycles, confirming the stability of the immobilization system.

Comparable immobilization performance has been reported for other alginate-based systems. Varga et al. [23] reported an immobilization efficiency of 98.20% for commercial laccase immobilized in alginate beads. Similarly, immobilization efficiencies of 92% and 89% were reported for commercial *T. versicolor* laccase immobilized in Cu-alginate and Ca-alginate beads, respectively [43]. The same study also showed approximately 68% residual activity after the third cycle [43], which is in good agreement with the operational stability observed in the present study.

To further elucidate the structural and surface properties of the immobilized biocatalyst, SEM analysis was performed.

The observed morphological changes after immobilization are likely associated with the interactions between the proteins present in the crude extracellular enzyme preparation and the alginate matrix, as well as with the glutaraldehyde activation step, which may induce modifications in the bead surface structure. The increased porosity observed in the immobilized beads may facilitate mass transfer by improving substrate diffusion to the catalytically active sites within the immobilized enzyme preparation [34].

The presence of crystalline structures on the surface of the immobilized beads may be attributed to residual ammonium sulfate originating from the enzyme precipitation step. Similar morphological characteristics of porous alginate matrices have previously been reported by Noreen et al. [42], who suggested that the porous structure of alginate beads may contribute to efficient enzyme immobilization and improved biocatalyst performance.

FT-IR spectroscopy was further employed to investigate the interactions between the crude extracellular enzyme preparation and the GA-activated alginate matrix following immobilization.

The broad absorption band observed in the free crude extracellular enzyme preparation (3200–3400 cm^−1^) is typically associated with NH_2_ group vibrations in proteins, while the peak at 3301 cm^−1^ in alginate corresponds to OH stretching vibrations. The disappearance of this peak after immobilization, together with the appearance of a new band at 3026 cm^−1^, suggests interactions between protein functional groups present in the crude extracellular laccase preparation and the alginate support, possibly involving the formation of new bonding environments during the immobilization process [44,45].

Further spectral changes, including the disappearance of peaks at 1265 cm^−1^ and 1169 cm^−1^, which are associated with C–N stretching and C–C–N bending vibrations, respectively, also indicate involvement of amine groups in interactions with the support matrix [45]. The shifts of the bands at 939 cm^−1^ and 884 cm^−1^ to higher wavenumbers further support structural modifications in the immobilized system. The appearance of a new band at 606 cm^−1^ may be related to amide-type vibrations formed during enzyme immobilization, further supporting the interactions between proteins present in crude extracellular laccase preparation and the GA-activated alginate beads [45].

To further assess the applicability of the developed free and immobilized crude extracellular laccase preparation in environmental biocatalysis, its efficiency in BPB decolorization was subsequently evaluated under varying reaction conditions.

Reports describing BPB decolorization by *B. adusta* laccase remain scarce. However, previous studies have demonstrated the ability of *B. adusta* to decolorize a wide range of azo, anthraquinone, phthalocyanine, and polyaromatic dyes [28,46]. In a previous study, free and immobilized crude laccase preparations from *B. adusta* TMF1 achieved more than 63% of decolorization of a mixture of azo dyes within 30 min [47].

Previous studies have also investigated BPB decolorization using laccases from *Paraconiothyrium variabile*, *Aspergillus oryzae*, and *T. versicolor* in the presence and absence of the synthetic mediator hydroxybenzotriazole (HBT) (Table 3) [48]. In the absence of HBT, *P. variabile* laccase decolorized less than 50% of BPB after 30 min, whereas approximately 90.6% decolorization was achieved after 3 h in the presence of HBT [48]. Laccases from *A. oryzae* and *T. versicolor* showed approximately 25.3% BPB decolorization after 3 h [48]. Compared with these systems, the crude extracellular laccase preparation from *B. adusta* TMF1 laccase achieved relatively high BPB decolorization within a short reaction time without the addition of synthetic mediators.

The improved performance of the immobilized crude extracellular laccase preparation, particularly its faster initial decolorization and higher overall decolorization efficiency (78.12% vs. 73.02% for free preparation), may be attributed to the enhanced stability of the catalytically active proteins and favorable microenvironment provided by the alginate matrix. The ability to achieve efficient decolorization under mediator-free conditions is particularly advantageous, as synthetic redox mediators may increase process complexity, operational cost, and environmental burden. Furthermore, comparison with previously reported fungal laccase systems indicates that the immobilized crude extracellular laccase preparation from *B. adusta* TMF1 achieved BPB decolorization efficiencies comparable to or higher than those reported for several free and immobilized fungal laccases under mediator-free conditions (Table 3). Although higher decolorization efficiencies have been reported for some fungal systems, such as *Ganoderma* sp. En3 (98.3% after 12 h) [49], these results were obtained after substantially longer reaction times. Similarly, immobilized laccase from *Ganoderma* sp. KU-alk4 achieved 64.4% decolorization only after 12 h [50]. In contrast, the immobilized *B. adusta* TMF1 laccase achieved 78.12% decolorization within 1 h without the addition of synthetic mediators. These findings further support the potential of the developed immobilized biocatalyst for rapid mediator-free BPB decolorization.

**Table 3:** Comparison of BPB decolorization by free and immobilized crude extracellular laccase preparation from *B. adusta* TMF 1 with selected fungal laccase systems.

Free Enzyme Preparations/Laccase Systems	Decolorization Efficiency of BPB (%)	Incubation Time (h)	Mediator	References
*B. adusta* TMF1	73.02	1	NO	Present study
*P. variabile*	72.2	0.5	NO	[51]
*P. variabile*	90.6	3	5 mM HBT	[51]
*A.oryzae*	25.3	3	5 mM HBT	[51]
*T. versicolor*	25.3	3	5 mM HBT	[51]
Immobilized enzyme preparations/laccase systems	Decolorization efficiency of BPB (%)	Incubation time (h)	Mediator	References
*B. adusta* TMF1	78.12	1	NO	Present study
*Ganoderma sp.* KU-alk4	64.4%	12	NO	[50]

Although the immobilized preparation retained catalytic activity over four consecutive decolorization cycles, a gradual decline in decolorization efficiency was observed. As enzyme leakage was negligible, the reduced reusability is more likely attributable to gradual enzyme inactivation during repeated catalytic cycles. This activity loss may be associated with prolonged exposure to dye molecules and their transformation products, oxidative stress generated during the catalytic process, conformational changes in the immobilized proteins, or partial blockage of active sites by adsorbed reaction products. In addition, repeated washing and prolonged incubation during successive reaction cycles may have further contributed to the gradual decline in catalytic efficiency despite the enzyme preparation remaining immobilized.

To further investigate the structural changes in BPB following enzymatic treatment, HPLC analysis was performed to monitor changes in the chromatographic profile after treatment with the crude extracellular laccase preparation from *B. adusta* TMF1.

The observed alterations in the chromatographic profiles, particularly the appearance of additional peaks at 254 nm in the treated samples, indicate the formation of transformation products following enzymatic treatment. The absence of major changes at 592 nm suggests that the parent chromophoric structure may not be completely eliminated under the tested conditions, despite significant decolorization observed spectrophotometrically.

Although these findings suggest the transformation of BPB by the crude extracellular laccase preparation, the exact nature of the generated products cannot be determined based on HPLC analysis alone. Therefore, more advanced analytical techniques such as LC-MS or GC-MS would be required to fully elucidate the degradation pathways and identify specific transformation products.

Considering the known toxicity of triphenylmethane dyes and their transformation products, further evaluation of the treated samples was necessary [46]. Therefore, additional phytotoxicity and antimicrobial assays were performed to assess whether enzymatic treatment leads not only to decolorization but also to a reduction in overall toxicity.

The phytotoxicity assay showed that BPB at a concentration of 50 mg/L did not exhibit pronounced phytotoxic effects toward *T. aestivum*, as reflected by the relatively high germination index. More importantly, enzymatic treatment further enhanced seed germination and root development, as evidenced by the increased GI value compared to the untreated sample. This suggests that enzymatic treatment with the crude extracellular laccase preparation reduced the phytotoxic effects of BPB toward *T. aestivum* under the tested conditions.

Information on the phytotoxicity of BPB and its transformation products remains limited. However, similar detoxification effects have been reported for structurally related triphenylmethane dyes. Li et al. [52] demonstrated that crystal violet and methyl violet significantly inhibited germination and root growth of *T. aestivum*, whereas enzymatic treatment substantially reduced their phytotoxic effects and restored root development to levels comparable to the control.

Although these findings indicate a reduction in phytotoxicity following enzymatic treatment, further ecotoxicological studies are required to comprehensively evaluate the environmental safety and potential application of the treated effluents.

Certain triphenylmethane dyes have been reported to exhibit antibacterial and antifungal properties [53]. In addition, both the parent dyes and their transformation products may affect microbial communities in aquatic environments, highlighting the importance of evaluating the potential antimicrobial effects of treated dye solutions [54].

The observed slight reduction in the viability of *C. albicans* in the presence of BPB, together with the absence of inhibitory effects on *L. rhamnosus*, is consistent with previous reports indicating that BPB exhibits limited antimicrobial activity at relatively low concentrations. Similar findings were reported for BPB concentrations between 40 and 400 mg/L, where no antimicrobial effects were observed against *Staphylococcus aureus* and *Klebsiella aerogenes* [55]. Likewise, another study demonstrated that higher BPB concentrations were shown to inhibit several *Lactobacillus* strains, whereas lower concentrations had no effect on bacterial growth [56].

Since the BPB concentration used in the present study (50 mg/L) is the below previously reported inhibitory levels, the absence of antimicrobial activity toward *L. rhamnosus* is consistent with the available literature. Importantly, enzymatic treatment eliminated the slight inhibitory effect observed for untreated BPB and did not negatively affect any of the tested microorganisms. The slight increases in viable cell numbers observed after treatment suggest that the transformation products generated during enzymatic treatment were not toxic to the tested strains under the experimental conditions employed. These findings are consistent with the reduced toxicity observed in the phytotoxicity assay and support the potential environmental compatibility of the laccase-treated samples.

## 5. Conclusions

This study presents the first report on the application of free and alginate-immobilized crude extracellular laccase preparation from *B. adusta* TMF1, produced on a wheat bran/barley bran mixture, for BPB decolorization without the addition of synthetic mediators. Both free and immobilized preparations demonstrated efficient decolorization, reaching 73.02% and 78.12%, respectively. In addition, the immobilized preparation exhibited good operational stability and reusability during repeated decolorization cycles.

HPLC analysis revealed changes in the chromatographic profile of BPB following enzymatic treatment, suggesting the formation of transformation products. Phytotoxicity and antimicrobial assays indicated reduced toxicity of the treated samples under the tested conditions. The treated samples showed no inhibitory effects toward the tested microorganisms and exhibited improved germination parameters in *T. aestivum* compared with untreated BPB.

The obtained results demonstrate the potential of the crude extracellular laccase preparation from *B. adusta* TMF1 as an environmentally friendly biocatalyst for BPB decolorization. Furthermore, the use of low-cost agro-industrial residues for enzyme production highlights the potential integration of this approach into sustainable wastewater treatment and biorefinery strategies. Future studies employing advanced analytical techniques, such as LC-MS/MS, would provide further insight into the transformation products generated during BPB treatment and their environmental relevance.

## Figures and Tables

**Table 1:** Michaelis–Menten kinetics parameters of free and immobilized crude extracellular laccase preparation for ABTS oxidation.

Biocatalyst	K_m_ (µM)	V_max_ (µM/min)	R^2^
Free crude extracellular laccase preparation	86	549	0.996
Immobilized crude extracellular laccase preparation	376	153	0.993

**Table 2:** Antimicrobial effects of BPB and enzymatically treated PBP samples on selected microorganisms.

Microorganism	Effect of BPB	Effect of Degraded BPB
*L. rhamnosus* ATCC 7469	-	2.12% ^b^
*C. albicans* ATCC 10259	5.17% ^a^	1.8% ^b^
*S. cerevisiae*	7.32% ^b^	8.77% ^b^

^a^—inhibition percentage, ^b^—stimulation percentage.

## Data Availability

Please add the corresponding content of this part. The data presented in this study are available from the corresponding author upon reasonable request.

## Supplementary Materials

The following supporting information can be downloaded at: https://www.mdpi.com/article/10.3390/jof12070531/s1. Figure S1: SDS-PAGE analysis of extracellular enzyme preparations obtained from *Bjerkandera adusta* TMF1. Lane M, protein molecular weight marker (25–250 kDa); lane 1, crude extracellular enzyme preparation; lane 2, protein fraction precipitated with 70% ammonium sulfate; lane 3, crude enzyme preparation obtained after ultrafiltration using a 50 kDa Amicon membrane. Figure S2: HPLC analysis of Bromophenol blue before and after enzymatic treatment with crude laccase from *B. adusta* TMF1. (a) Detection at 592 nm. (b) Detection at 254 nm. Figure S3: Phytotoxicity test of Bromophenol Blue and laccase-treated samples toward *Triticum aestivum*. (a) Control, (b) Bromophenol Blue, and (c) laccase-treated Bromophenol Blue.

## Abbreviations

The following abbreviations are used in this manuscript:

AAO	aryl alcohol oxidase
ABTS	2,2′-azino-bis(3-ethylbenzothiazoline-6-sulfonic acid
BB	barely bran
BPB	Bromophenol Blue
BSA	bovine serum albumin
DyPs	dye decolorizing peroxidase
FT-IR	Fourier transform infrared spectroscopy
GA	glutaraldehyde
GI	germination index
HBT	hydroxybenzotriazole
HPLC	high-performance liquid chromatography
IE	immobilization efficiency
LiP	lignin peroxidase
MnP	manganese peroxidase
MRS	de Man Rogosa and Sharpe
RGV	relative germination value
RLV	relative root length value
SSF	solid state fermentation
SmF	submerged fermentation
TSB	tryptic soy broth
VP	versatile peroxidase
WB	wheat bran
