# Supplementary material for: Decolorization of Bromophenol Blue by Free and Immobilized Crude Extracellular Laccase Preparation from Bjerkandera adusta TMF1 Produced on Agro-Industrial Residues
Source: J Fungi (Basel). 2026 Jul 19;12(7):531. doi: 10.3390/jof12070531 (PMC13412901; doi:10.3390/jof12070531)
Supplement: Supplementary file 1 [file jof-12-00531-s001.zip › jof-4392974-supplementary.pdf]

Supplementary material

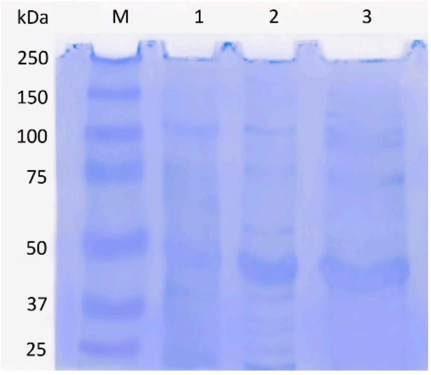

Figure S1 SDS-PAGE analysis of extracellular enzyme preparations obtained from *Bjerkandera adusta* TMF1. Lane M, protein molecular weight marker (10–250 kDa); lane 1, crude extracellular enzyme preparation; lane 2, protein fraction precipitated with 70% ammonium sulfate; lane 3, crude laccase preparation obtained after ultrafiltration using a 50 kDa Amicon membrane.

a)

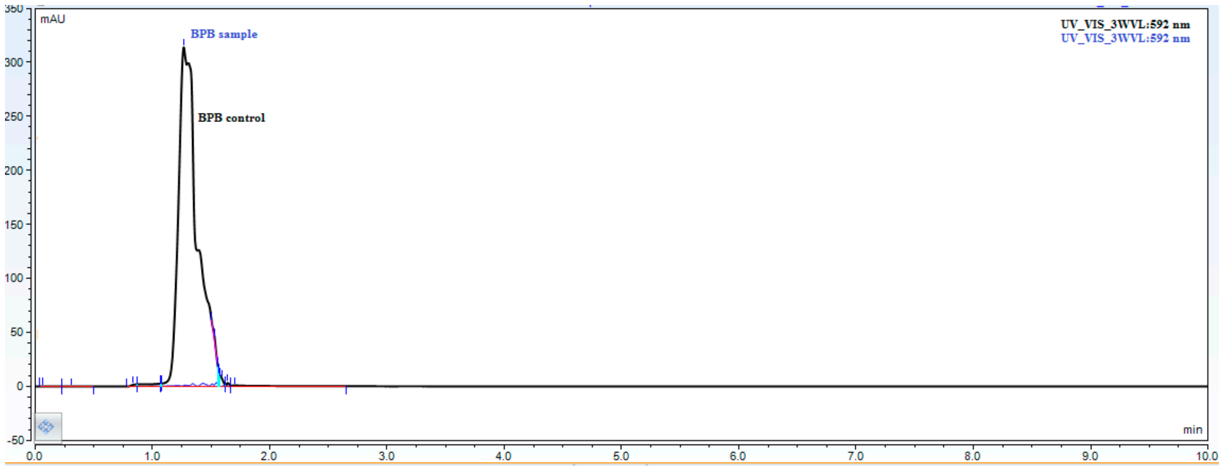

b)

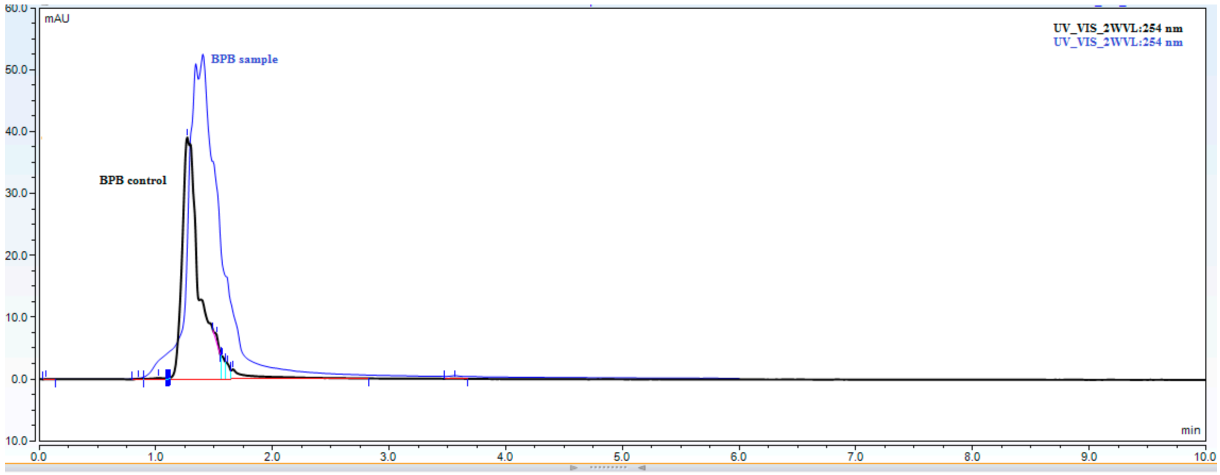

Figure S2. HPLC analysis of Bromophenol blue and its degraded products before and after decolorization with crude fungal laccase, a) HPLC analysis of Bromophenol blue and its degraded products at 592 nm; b) HPLC analysis of Bromophenol blue and its degraded products at 254 nm.

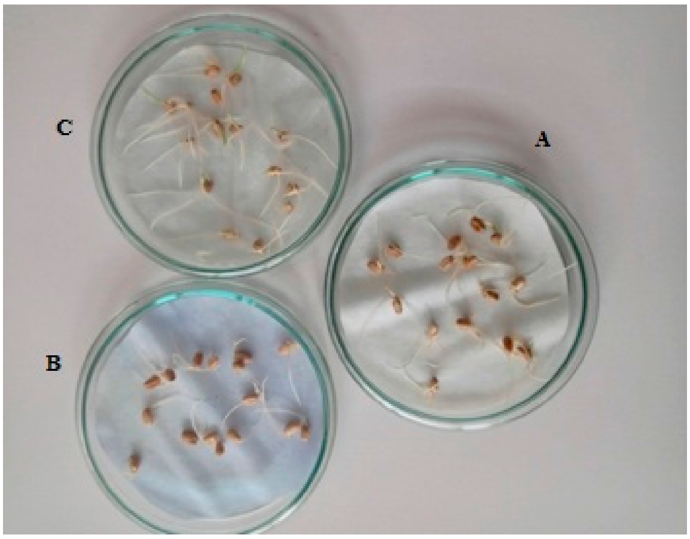

Figure S3. Phytotoxcity test of Bromophenol blue and its degraded products. The effect of Bromophenol blue and its degraded products on the seeds germination of *T. aestivum*, **A**) control, **B**) Bromophenol blue, **C**) degraded products of dye.
